# Supplementary material for: Are Supplements Safe? Effects of Gallic and Ferulic Acids on In Vitro Cell Models
Source: Nutrients. 2020 May 29;12(6):1591. doi: 10.3390/nu12061591 (PMC7352663; doi:10.3390/nu12061591)
Supplement: Supplementary file 1 [file nutrients-12-01591-s001.zip › supplementary_data/Supplementary data_Nutrients.docx]

**Supplementary Materials and Methods**

**Cell model systems**

NCM460 normal human colon mucosal epithelial cell line were cultured with DMEM, to which 10 % fetal bovine serum, 1mM L-glutamine, 1 % penicillin-streptomycin and 1 % sodium pyruvate (GIBCO, Massachusetts, USA) were added.

**Intestinal equivalents**

For intestinal equivalents, 0.5 ml of a cell free collagen solution (1.35 mg/ml rat tail collagen type I in DMEM with 10% FCS and 1% Pen/Strep) was added to tissue culture inserts (Transwell, Costar, Cambridge, MA) in 12-well plates. This pre-coated layer was overlaid with 1ml of L929 fibroblasts (10^5^/ml) together with monocytes (3*10^4^/ml) mixed with collagen type I. After 2 hours of incubation at 37°C, 2*10^5^ of NCM460 cells were seeded on dermal reconstructs and incubated at 37°C with NCM460 medium, added both on the upper and in the lower part of the filter support. After 5 days, intestinal equivalents were either treated or not treated with 5 or 20 mg/L of either gallic or 70% EtOH for the control. The intestinal equivalents were then fixed with formalin for 2 hours at room temperature, dehydrated and embedded in paraffin.

**Supplementary Results**

**
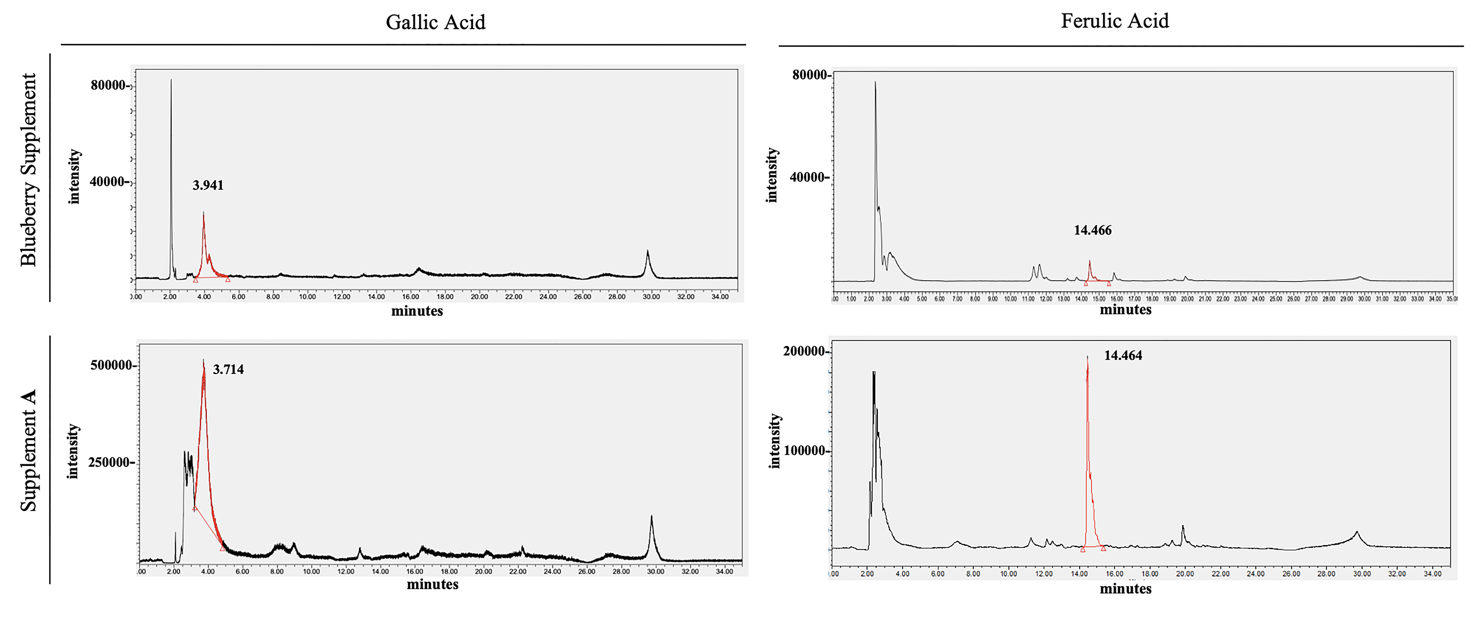
**

**Supplementary Figure 1.** Chromatograms obtained with HPLC-MS/MS analysis of the gallic and ferulic acid contents in both Blueberry Supplement and Supplement A.

**
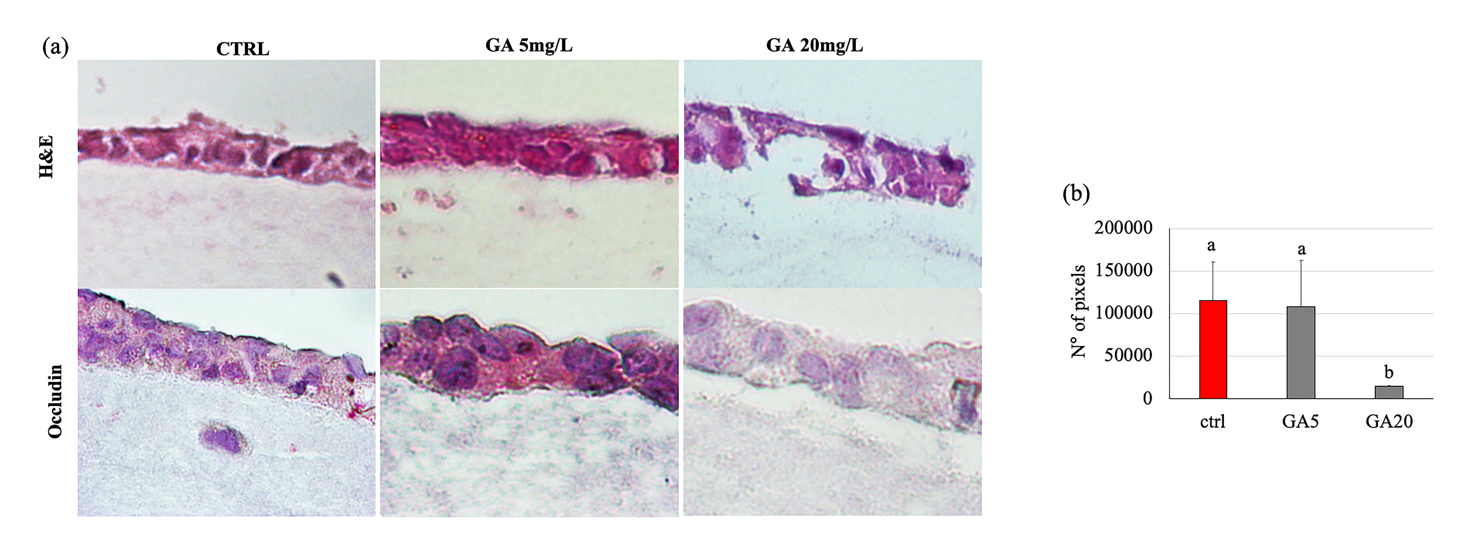
**

**Supplementary Figure 2.** Intestinal equivalents obtained by seeding NCM460 cells on dermal equivalents that were paraffin-embedded. (**a**) Sections were stained with hematoxylin and eosin (H&E), and occludin marker; fast red was used as a chromogen. (**b**) Stained areas were evaluated by image pixel count using ImageJ 2.0.0-rc-69/1.521. Experiments were conducted in triplicate from different samples. Statistical analysis was performed and between brackets, different letters indicate mean values that are significantly different at P <0.05.
