# Supplementary figures and images for: Are Supplements Safe? Effects of Gallic and Ferulic Acids on In Vitro Cell Models
Source: Nutrients. 2020 May 29;12(6):1591. doi: 10.3390/nu12061591 (PMC7352663; doi:10.3390/nu12061591)

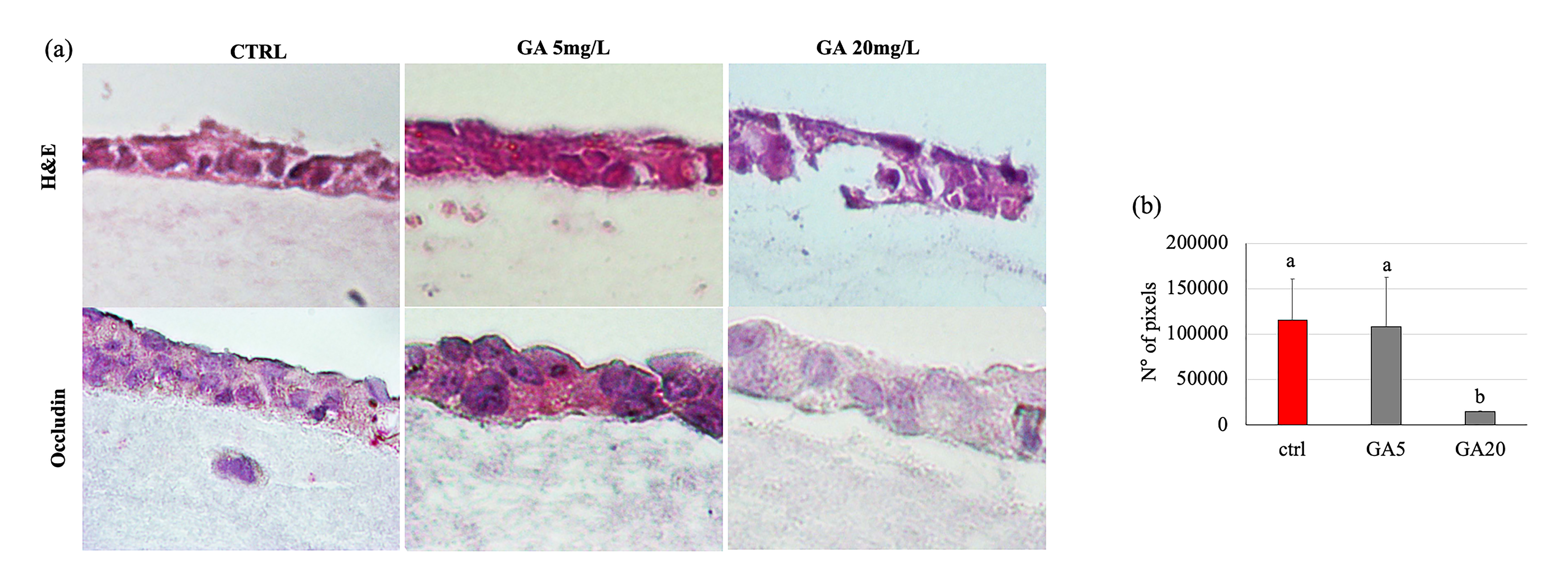

Supplement: Supplementary file 1 [file nutrients-12-01591-s001.zip › supplementary_data/Supplementary_Fig_2.tif]
